# Exploring the frontiers: a panoramic analysis of global multi-specific antibody clinical trials

**DOI:** 10.1097/JS9.0000000000002329

**Published:** 2025-03-14

**Authors:** Simeng Gao, Yan Zhang, Jingru Han, Jianfu Zhao

**Affiliations:** Research Center of Cancer Diagnosis and Therapy, Department of Oncology, The First Affiliated Hospital of Jinan University, Guangzhou, China

**HIGHLIGHTS**

- PsAbs enhance targeting efficacy in tumor therapy.
- Multi-antibody strategy may reduce dosage and side effects.
- 3.Combination therapies like trastuzumab and pertuzumab show superior outcomes.

*Dear Editor,*

Multi-specific antibodies are immunoglobulins that can bind to two or more distinct epitopes or antigens simultaneously, thereby augmenting the specificity and efficacy of targeting tumor cells^[^1^]^. In comparison to monoclonal antibody therapy, the multi-specific antibody therapy has several advantages. Notably, it extends the duration of therapeutic efficacy by using a multi-targeting strategy, which reduces the possibility of tumor cells to develop resistance to all administered agents concurrently^[^2^]^. Secondly, by concurrently administering multiple antibodies, the dosage of individual antibodies can be reduced, thereby mitigating the associated adverse effects^[^3^]^. Thirdly, the multispecific antibody therapy attenuates the cytotoxic impact on normal cells by distributing the therapeutic burden, consequently enhancing the quality of life of patients^[^4^]^. At present, multi-antibody therapy has significant clinical advancements. For instance, combination antibody therapies for HER2-positive (human epidermal growth factor receptor 2) breast cancer, such as the co-administration of trastuzumab and pertuzumab, have superior therapeutic outcomes^[^5^]^. More and more researchers are investing in the study of tumor multi-specific antibody therapy. Therefore, we analyzed the current clinical trials of multi-specific antibody around the world to provide the necessary data analysis for clinical therapies of cancer and the relevant regulatory authorities.

We conducted a comprehensive query of the Informa database (https://pharma.id.informa.com/) using the search parameters “therapeutic category: anticancer, multispecific antibodies” and “therapeutic area: oncology.” This search yielded a total of 1274 clinical trials involving multispecific antibodies as of August 2024. The extracted data encompassed clinical trial names, types, targets, co-partners, and pertinent clinical information. Subsequently, we performed an in-depth analysis of trends in the number, proportion, types, clinical trial phases, clinical indications, and other relevant parameters of multi-specific antibodies clinical trials to evaluate global trends in this domain.

From 1996 to 2024, 1274 clinical trials of multi-specific antibody therapy (Supplemental Digital Content, http://links.lww.com/JS9/E11) were planned worldwide, mainly in 18 countries. Of these trials, China led the way with 702 trials, with more research activity in this area than any other country, followed by the United States, with 381 relevant trials conducted (Fig. 1A). Notably, the number of these clinical trials has a significant increasing trend since 2016 (Fig. 1B), which may reflect the growing global recognition of the therapeutic potential of multispecific antibodies and increased investment in their research. Among these clinical trials, Phase II/III trials dominated, whereas only a few trials progressed to Phase IV, indicating that current research on multi-specific antibody therapy was at a critical stage of clinical development. In addition, these trials covered almost all types of tumors, mainly solid tumors. Most of them focused on first-line to second-line treatments, reflecting the high clinical reliance on this type of therapeutic strategy (Fig. 1C).

**Figure 1.**
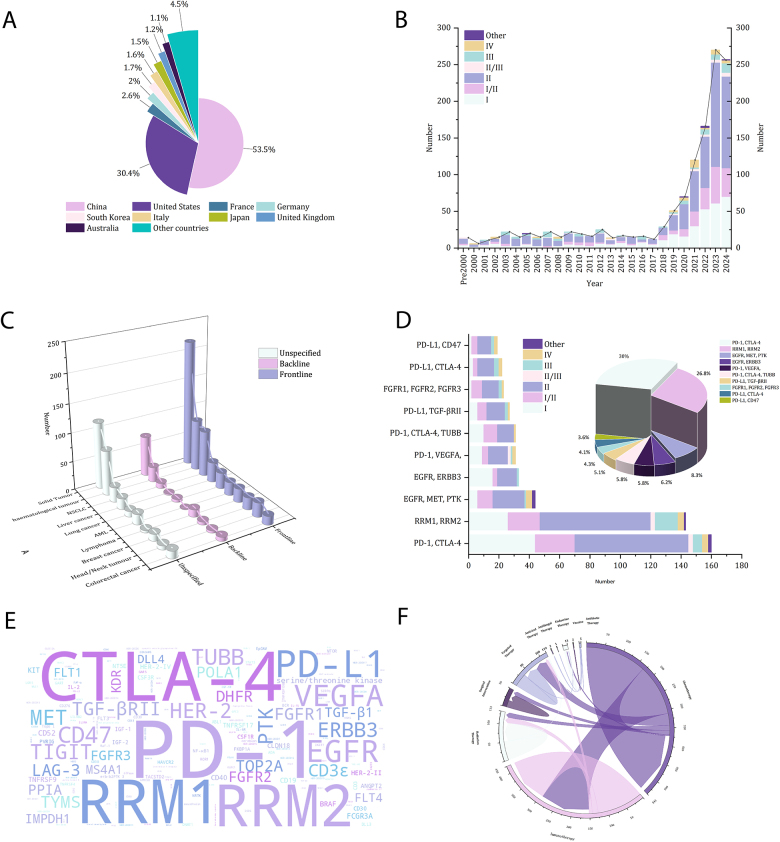
Distribution of studies of multi-specific antibody therapies in cancer. (A) Distribution of phases of clinical trials of multi-specific antibody therapies by year. (B) Distribution of cancers and lines of treatment of patients receiving multi-specific antibody therapies. (C) Top 10 target combinations along with the distribution of phases of clinical trials. (D) Combination regimens of multi-specific antibody therapies and other therapies. (E) Multi-specific antibody therapeutic target cloud map. (F) Global breakdown of clinical trials of multi-specific antibody therapies respectively.

Further analysis of the top 10 multi-specific antibody target combinations showed that bispecific antibody combinations were the most common, with the number one combination of CTLA-4 and PD-1 (30%), followed by the combination of RMM1 and RMM2 (26.8%), and the highest three-target combination was the combination of EGFR, MET, and PTK (8.3%). Most of these combinations have appeared in Phase II and Phase III clinical trials, demonstrating their potential in cancer immunotherapy, especially in combination immunotherapy and multi-target synergy. The selection of these target combinations reflects their promising broad application in overcoming immune escape, boosting immune response and improving efficacy. These were still dominated by Phase II/III clinical trials, followed by Phase I clinical trials (Fig. 1D). (Table 1). Analysis of the targets studied in all clinical trials showed that PD-1 appeared most frequently, followed by CTLA-4 (Fig. 1E). This finding not only highlights the centrality of immune checkpoint inhibitors in current cancer treatment strategies, but also points to possible directions for future therapeutic innovation.

**Table 1:** Partial Clinical Trial Information.

Trial ID	Trial title	Trial phase	Trial status	Tumor	Patient segment	Primary tested drug	Target	Years	Countries
389044	A Phase I/II Study Evaluating the Safety and Efficacy of Amivantamab and Capmatinib Combination Therapy in Unresectable Metastatic Non-small Cell Lung Cancer	I/II	Closed	Solid tumor	(N/A); DIPG; Second line	Cadonilimab	CTLA-4, PD-1	2023	China
213218	A Phase I/II, Open Label, Study of Amivantamab (JNJ-61186372) Among Participants With Advanced NSCLC Harboring ALK, ROS1, and RET Gene Fusions in Combination With Tyrosine Kinase Inhibitors	I/II	Open	Solid tumor	Adenocarcinoma; Advanced; Classical; Extensive; First line; High-grade; Metastatic; MSI-H/dMMR; Nodular lymphocyte-predominant; PD-1 Naive; PD-1 Refractory; PD-L1 Naive; PD-L1 Refractory; Pulmonary; Second line; Squamous Cell; Stage III; Stage IV; Unresectable; Untreated	Vudalimab	CTLA-4, PD-1	2022	United States
72142	Reduced Intensity Haploidentical Hematopoietic Stem Cell Transplantation (HSCT) Supplemented With Donor Natural Killer (NK) Cell Infusions in Patients With High Risk Myeloid Malignancies Who Are Unsuitable for Fully Myeloablative Transplantation	I/II	Completed	Soft Tissue Sarcoma	Metastatic; Second line	Fludarabine (IV) allogeneic stem cells thymoglobulin	RRM1, RRM2	2007	Japan
233676	A Pilot Trial of Reduced Intensity Allogeneic Stem Cell Transplantation With Fludarabine, Melphalan, and Low Dose Total Body Irradiation	II	Completed	Haematological tumour	Accelerated phase; Aggressive; Chronic phase; Classical; Extranodal marginal zone B-cell lymphoma (MALT); Follicular lymphoma (FL); High risk; Indolent; Int-2 risk; Mantle cell lymphoma (MCL); Nodular lymphocyte-predominant; Other subtype; Peripheral T-cell lymphoma (PTCL); Poor-risk; Post-transplant/ineligible; Primary myelofibrosis; RAEB; RAEB-t; RCMD; Remission; Second line; Small lymphocytic lymphoma (SLL); Stage I; Stage II; Stage III; Stage IV	Fludarabine (IV) melphalan allogeneic stem cells undisclosed—anti-thymocyte globulin	RRM1, RRM2	2006	United States
499546	An Open, Multicenter, Phase I Clinical Study to Evaluate the Safety, Tolerability, Pharmacokinetics/Pharmacokinetics, and Antitumor Activity of GNC-038 Quad-specific Antibody Injection in Relapsed or Refractory Non-Hodgkin’s Lymphoma, Relapsed or Refractory Acute Lymphoblastic Leukemia, and Refractory or Metastatic Solid Tumors	I	Open	Haematological tumour	(N/A); Accelerated phase; Aggressive; Blast phase; Chronic phase; Classical; Indolent; Nodular lymphocyte-predominant; Second line	Melphalan undisclosed—anti-thymocyte globulin busulfan, Otsuka fludarabine	RRM1, RRM2	2019	United States
495760	Lipid Core Nanoparticles as Vehicle for Etoposide in the Conditioning Regimen of Marrow Transplantation in Acute Myeloid Leukemia Not Responding to Induction Therapy: A Pilot Study.	II	Completed	Lung	EGFR; Second line; Stage III; Stage IV	PM-1080	EGFR, MET, PTK	2023	China
491153	A phase II study of KN046 monotherapy or in combination with chemotherapy in locally advanced unresectable or metastatic NSCLC.	II	Planned	Solid Tumor	EGFR; HER2 positive; MET Amplification/Alteration; Second line; Stage III; Stage IV	Amivantamab	EGFR, MET, PTK	2021	United States
482599	Reduced Toxicity, Myeloablative Conditioning Regimen with Busulfan, Fludarabine, Anti-Thymocyte Globulin and 400 Cgy TBI in Pediatric Patients Undergoing Hematopoietic Stem Cell Transplant for High-Risk Hematologic Malignancies	IV	Completed	Thymus	PD-1 Refractory; PD-L1 Refractory; Second line; Stage III; Stage IV; Third line; Thymic carcinoma	Erfonrilimab	PD-L1, CTLA-4,	2021	United States
482062	A Phase Ib/II Study Investigating Safety, Tolerability, Pharmacokinetics and Preliminary Antitumor Activity of Anti-HER2 Bispecific Antibody ZW25 in Combination With Chemotherapy With/Without Tislelizumab in Patients With Advanced HER2-positive Breast Cancer or Gastric/Gastroesophageal Junction Adenocarcinoma	I/II	Completed	Solid Tumor	Line of therapy N/A; Stage III; Stage IV	Erfonrilimab	PD-L1, CTLA-4,	2024	China

In addition, 643 clinical trials of multi-specific antibodies were conducted in combination with other treatment modalities, covering chemotherapy, targeted therapy, radiotherapy, surgery, immunotherapy, antiviral therapy, antibiotic therapy, endocrine therapy, and vaccine therapy (Fig. 1F). The top three combination treatment modalities were chemotherapy (169 cases, 26.2%), combined immunotherapy and chemotherapy (141 cases, 21.9%), and immunotherapy (127 cases, 19.8%). This extensive exploration of multimodal combination therapy strategies reflects the continuous clinical quest for improved therapeutic efficacy and also indicates the important role that multi-specific antibodies may play in the future of comprehensive cancer therapy. In recent years, the combination of immune checkpoint inhibitors (e.g., PD-1/PD-L1 inhibitors) with proprotein convertase subtilisin/kexin type 9 (PCSK-9) inhibitors has attracted attention. Studies have shown that inhibition of PCSK-9 can improve the efficacy of anti-PD-1/PD-L1 immunotherapy by improving immune cell function and potentially overcoming resistance to therapy^[^6,7^]^. This emerging strategy may provide new clinical benefits for immunotherapy-resistant cancer patients.

Multi-specific antibodies are a class of antibodies that can recognize and bind to multiple antigens or antigenic epitopes at the same time, offering significant advantages over traditional monoclonal antibodies. Due to its multi-targeting effect, it can target multiple tumor epitopes or immune cells simultaneously, thereby enhancing the immune response and therapeutic effect. For example, in cancer immunotherapy, multi-specific antibodies can simultaneously target immune checkpoints, such as PD-1 and CTLA-4, to exert a synergistic effect, promote stronger immune activation, and increase tumor clearance rates^[^8^]^. In addition, by virtue of multi-targeting, multi-specific antibodies are able to overcome problems of antigenic variation or immune escape, which often limit the efficacy of monoclonal antibodies. Compared to monoclonal antibodies, multi-specific antibodies activate a broader range of immune pathways, such as antibody-dependent cell-mediated cytotoxicity (ADCC) and T-cell activation, providing a more comprehensive immune response^[^9^]^. In the face of immune escape or drug resistance problems, multispecific antibodies offer greater adaptability and long-lasting therapeutic effects, thus having great potential in the treatment of complex diseases such as cancer.

The document titled “Guidance on Procedures for the Development of Polyspecific Antibodies,” issued by the U.S. Food and Drug Administration (FDA) in May 2021, offers comprehensive directives on the development of multi-specific antibodies. It encompasses a broad spectrum of regulatory, quality, non-clinical, and clinical considerations, all designed to ensure the safety and efficacy of the associated pharmaceutical products. In June 2022, China’s National Medical Products Administration (NMPA) granted approval for Cadonilimab, a PD-1/CTLA-4 bispecific antibody, for the treatment of recurrent or metastatic cervical cancer. This approval marks the world’s first bi-immune checkpoint inhibitor of tumor to be authorized for marketing and represents the first bispecific antibody drug developed in China. Although the multi-specific antibody market in China is currently in its nascent stages, the presence of numerous drug candidates undergoing clinical trials suggests that the market is poised for substantial growth in the forthcoming years. The policy initiatives in countries such as China and the United States indicate that the research and application of multi-specific antibody therapeutics are becoming a central focus in global pharmaceutical research and development. The advancement of this therapeutic strategy is expected to bring more efficacious treatments to cancer patients, while simultaneously serving as a significant indicator of progress in contemporary pharmaceutical science and technology.

We anticipate that multi-specific antibody therapies will be widely used in clinical practice, especially in addressing cancer types that are more challenging to treat with conventional therapies, with the development of individualized treatment and precision medicine. Currently, combination immunotherapy is an important research direction aimed at improving efficacy by integrating multiple immunotherapies to more precisely target and eradicate tumor cells. Because of the complex mechanism of action of multi-specific antibodies, which may trigger unexpected immune responses or other adverse reactions, their safety and efficacy need to be evaluated through extensive clinical trials. Meanwhile, compared with monoclonal antibodies, the design and production of multi-specific antibodies are more complex, which may lead to increased production costs. Therefore, to promote their commercialization and clinical translation, we need to optimize the production process, reduce costs, and enhance the implementation of clinical trials to verify their effectiveness in different patient populations. In addition, by establishing an efficient regulatory approval pathway, it will help to shorten the time from research and development to clinical application, accelerate its marketing, and ultimately lead to wider clinical application (Supplemental Digital Content, http://links.lww.com/JS9/E10).

## Data Availability

The data used in this study were sourced from the Informa database, accessible via the following link: https://pharma.id.informa.com/. This database provides extensive data related to the pharmaceutical industry. The data referenced in our study are openly accessible to all interested individuals or institutions through the aforementioned link.
